# Mental Wellbeing and Quality of Life Among Patients With Diabetes Suffering From Hypoglycaemia in Saudi Arabia

**DOI:** 10.7759/cureus.17586

**Published:** 2021-08-30

**Authors:** Emad Salawati

**Affiliations:** 1 Family Medicine, King Abdulaziz University Faculty of Medicine, Jeddah, SAU

**Keywords:** dm, hypoglycaemia, mental wellbeing, emotional distress, quality of life

## Abstract

Background

Diabetes mellitus is a global burden that is considered a major public health concern for many countries. Saudi Arabia is ranked second among the highest percentages of diabetes worldwide, with more than 7 million patients with diabetes reported in 2017, with an estimated prevalence to be around 14%. Psychological and mental health outcomes are poorer in patients with diabetes who are suffering from hypoglycaemia. The aim of the study was to investigate the mental wellbeing and quality of life of patients with diabetes suffering from hypoglycaemia in Saudi Arabia.

Methods

A cross-sectional study using an online self-administered questionnaire was conducted between the 13^th^ of June and the 19^th^ of July 2021 in Saudi Arabia.

Results

A total of 69.7% of the study participants scored below 13 on the WHO-5 scale, which indicates poor mental wellbeing status and possible depression, and reduced quality of life. A total of 67.8% of the study participants scored equal to or greater than 8 on the PAID-5 scale, which indicates possible diabetes-related emotional distress that warrants further assessment.

The majority of the study participants (83.4%) scored equal to or greater than 28 on the fear of hypoglycaemia scale, which is classified as having fear of hypoglycaemia.

Conclusion

Depressive symptoms and reduced quality of life are common among patients with diabetes mellitus suffering from hypoglycaemia in Saudi Arabia.

## Introduction

Diabetes mellitus (DM) is a highly prevalent disease in Saudi Arabia and worldwide. Moreover, DM is considered a major public health concern for many countries in the world [1]. According to the World Health Organization (WHO), Saudi Arabia is ranked second among the highest percentages of diabetes worldwide, with more than 7 million patients with diabetes reported in 2017 [2], with the prevalence being estimated to be around 14% [1]. Patients with diabetes are usually treated with pharmacological treatment to control their blood sugar levels, and this put them at higher risk of developing some life-threatening and costly complications such as hypoglycaemia [3-5].

Hypoglycaemia is defined as low level of blood glucose in the body [3]. Hypoglycaemia is a major limitation of diabetes treatment, and it is usually acute in its nature [6]. The prevalence of hypoglycaemia has increased in the last years [7], and more attention in research and clinical practice is being focused on this issue [8]. A systematic review and meta-analysis on the incidence and prevalence of hypoglycaemia reported that the prevalence of hypoglycaemia ranged from 0.074% to 73.0% [8]. Another systematic review and meta-analysis reported that the prevalence of hypoglycaemia was 45% (95%CI 0.34,0.57) for mild/moderate and 6% (95%CI, 0.05,0.07) for severe cases [9].

Hypoglycaemia can be mild or severe, causing serious complications such as cardiac mortality, dementia, seizures, fall-related fractures and death [6,10-12]. These complications can be a major issue in the management plan of patients with diabetes due to reasons such as fear of hypoglycaemia episodes and avoidance of treatment [13]. The quality of life of patients suffering from hypoglycaemia is affected due to reasons such as social and behavioural embarrassment [14,15]. In addition, previous studies have reported that the psychological and mental health outcomes are poorer in patients with diabetes who are suffering from hypoglycaemia [16]. These studies also highlighted a variation in outcomes and associations at the country level and the need for further research in this area.

Previous studies investigating the mental burden and the effect on quality of life of hypoglycaemia in patients with diabetes are limited with few studies that mainly focused on the elderly population [16]. However, in recent years, more younger patients are diagnosed with both types of diabetes [17-19]. In addition, no previous studies have investigated this issue in the Middle East. Therefore, this study aimed to investigate the mental wellbeing and the quality of life of patients with diabetes and suffering from hypoglycaemia in Saudi Arabia.

Objectives

The primary objective of the study was to investigate the mental wellbeing and quality of life of patients with diabetes suffering from hypoglycaemia in Saudi Arabia. Secondary objectives were to explore diabetes-related emotional distress and the level of fear of hypoglycaemia among patients with diabetes in Saudi Arabia.

## Materials and methods

Study design

A cross-sectional study using an online self-administered questionnaire was conducted between the 13th of June and the 19th of July 2021 in Saudi Arabia. 

Sampling strategy

Convenience sampling techniques were used to recruit the study participants. Patients with diabetes mellitus who were aged 18 years and above and who were using antidiabetic therapy were invited to participate in the study. These inclusion criteria were highlighted in the invitation letter that was sent along with the study survey link. Social media platforms such as WhatsApp, Twitter, and Facebook were used to recruit the study participants. 

Questionnaire tool

Three previously validated assessment scales were used in this study [20-23]. The questionnaire was translated into Arabic and validated by three experts in the field. The World Health Organization (WHO)-5 measure of mental wellbeing (five items scale) [20] measures the degree of positive mental well-being in the past two weeks. Each item is scored using a six-point Likert scale that ranges from zero (not present) to five (constantly present). The total possible score for this scale ranges from 0 to 25, where scores below 13 indicate poor mental wellbeing and possible depression and reduced quality of life [20]. Problem Areas in Diabetes Scale (PAID-5) (five items scale) [21,22] scale measures diabetes-related emotional distress that warrants further assessment. Each item is scored using a five-point Likert scale that ranges from zero (not a problem) to four (serious problem). The total possible score for this scale ranges from 0 to 20, where scores equal to or greater than eight indicates possible diabetes-related emotional distress that warrants further assessment [21,22]. 15-item Fear of Hypoglycaemia scale (FH-15) [23] measures diabetic patients fear of hypoglycaemia using a five-point Likert scale that ranges from one (strongly disagree) to five (strongly agree). The total possible score for this scale ranges from 15 to 75, where scores equal to or greater than 28 would be classified as having fear of hypoglycaemia [23].

Statistical analysis

Data were analysed using Statistical Package for Social Science (SPSS) software, version 27 (IBM Corp, Armonk, NY, USA). Categorical variables were reported as frequencies and percentages. Significant predictors of poor mental wellbeing, problem areas in diabetes, and fear of hypoglycaemia were determined using binary logistic regression. A confidence interval of 95% (P < .05) was applied to represent the statistical significance of the results, and the level of significance was predetermined as 5%.

## Results

A total of 603 participants were involved in this study. More than half of them (56.2%) were females while a similar percentage (56.5%) were aged 35 years and below. A total of 65.8% are married. Sixty per cent of them reported that they are holding college/university degree or above. Around 44.0% of them reported that they are employed. The majority of them (72.1%) reported that they have type 2 diabetes mellitus. Around 40.0% of them reported that they have diabetes for a duration of five years or below. Around 44.0% of the study participants reported they are using oral antidiabetic therapy. Less than half of the study participants (45.9%) reported that they were admitted to the hospital for hypoglycaemic episodes in the previous six months. However, more than half of them (54.9%) reported experiencing a drop in the blood sugar level but did not require them to go to the hospital and were able to deal with it alone or with the help of those close to them. The majority of the study participants (85.1%) reported that they experience hypoglycaemic events at different frequencies. For further details on the participants’ demographic characteristics, refer to Table 1.

**Table 1:** Participants demographic characteristics.

Demographics	Overall (n = 603)
Gender No. (%)	
Female	339 (56.2%)
Age No. (%)	
18-25 years	105 (17.4%)
26-30 years	122 (20.2%)
31-35 years	114 (18.9%)
36-40 years	64 (10.6%)
41-45 years	51 (8.5%)
46-50 years	42 (7.0%)
51 years and over	105 (17.4%)
Marital status No. (%)	
Married	397 (65.8%)
Educational level No. (%)	
Not educated	44 (7.3%)
Completed primary or lower	30 (5.0%)
Completed secondary grade	167 (27.7%)
College/university or above	362 (60.0%)
Employment status No. (%)	
Unemployed or retired	239 (39.6%)
Employed	264 (43.8%)
Student	100 (16.6%)
Type of diabetes mellitus No. (%)	
Type 1 diabetes mellitus	168 (27.9%)
Type 2 diabetes mellitus	435 (72.1%)
Duration of the disease No. (%)	
Less than one year	76 (12.6%)
Between 1 and 5 years	165 (27.4%)
Between 6 and 10 years	160 (26.5%)
Between 11 and 15 years	93 (15.4%)
More than 15 years	109 (18.1%)
Diabetes medication regimen No. (%)	
Oral antidiabetic medications	266 (44.1%)
Insulin	164 (27.2%)
Combination (Oral medications and insulin)	121 (20.1%)
Combination (oral medications and other injectable antidiabetic agents)	52 (8.6%)
Hospital admission for hypoglycaemic episodes in the previous 6-months No. (%)	
Yes	277 (45.9%)
Did the patient experience a drop in the blood sugar level, but did not require him to go to the hospital and was able to deal with it alone or with the help of those close to him? No. (%)	
Yes	331 (54.9%)
How often do you experience a drop in your blood sugar level? No. (%)	
Never	90 (14.9%)
Rarely	163 (27.0%)
Sometimes	208 (34.5%)
Often	113 (18.7%)
Nearly every day	29 (4.8%)

Measure of mental wellbeing

A total of 69.7% of the study participants scored below 13 on the WHO-5 scale, which indicates poor mental wellbeing status and possible depression and a reduced quality of life. Table 2 below highlights the mental health status of the study participants over the last two weeks’ period. A small percentage of the study participants (ranged between 2.6% to 4.5%) reported that they are free from any mental wellbeing problem. On the other hand, almost double this percentage (ranged between 7.6% to 10.1%) of the study participants reported having mental wellbeing problems all the time. 

**Table 2:** Measures of mental wellbeing for the study participants.

Over the last two weeks	All the time	Most of the time	More than half of the time	Less than half of the time	Some of the time	At no time
I have felt cheerful and in good spirits	4.2%	17.0%	15.1%	30.0%	25.5%	8.3%
I have felt calm and relaxed	2.6%	15.4%	14.6%	30.0%	27.4%	10.1%
I have felt active and vigorous	3.8%	12.1%	12.3%	28.9%	33.1%	9.7%
I woke up feeling fresh and rested	3.8%	13.5%	13.7%	28.2%	33.1%	7.6%
My daily life has been filled with things that interest me	4.5%	14.7%	15.9%	27.2%	28.9%	8.7%

Problem Areas in Diabetes Scale (PAID-5)

A total of 67.8% of the study participants scored equal to or greater than 8 on the PAID-5 scale, which indicates possible diabetes-related emotional distress that warrants further assessment. Table 3 below highlights the current diabetes problems among the study participants. The most commonly reported serious problems were coping with complications of diabetes and feeling that diabetes is taking up too much of the mental and physical energy every day, with 19.9% and 18.1%, respectively. On the other hand, 17.6% of our study participants reported that they do not feel depressed when they think about living with diabetes. 

**Table 3:** Current diabetes problems among the study participants (PAID-5 scale).

	Not a problem	Minor problem	Moderate problem	Somewhat serious problem	Serious problem
Feeling scared when you think about living with diabetes	14.3%	21.5%	28.0%	29.0%	7.2%
Feeling depressed when you think about living with diabetes	17.6%	19.3%	30.8%	22.8%	9.5%
Worrying about the future and the possibility of serious complications	10.9%	14.8%	28.9%	28.0%	17.4%
Feeling that diabetes is taking up too much of your mental and physical energy every day	15.3%	16.9%	29.2%	20.6%	18.1%
Coping with complications of diabetes	9.5%	18.2%	30.2%	22.2%	19.9%

Fear of hypoglycaemia

The majority of the study participants (83.4%) scored equal to or greater than 28 on the fear of hypoglycaemia scale, which is classified as having fear of hypoglycaemia. Table 4 below highlights the fear of hypoglycaemia among the study participants. The most commonly reported fear problem reported by the study participants was worrying about losing consciousness due to hypoglycaemia (35.9%) followed by fearing not recognizing the symptoms of hypoglycaemia (33.2%). The study participants reported a similar percentage of agreement regarding avoidance problems related to hypoglycaemia (avoiding social situations (meetings, outings, etc. due to fear of having a hypoglycaemic episode, afraid of taking a trip/holiday for fear of experiencing hypoglycaemia and stopping doing things used to do for fear of having a hypoglycaemic episode). 

**Table 4:** Assessment of fear of hypoglycaemia.

Fear	Strongly disagree	Disagree	Neutral	Agree	Strongly agree
Are you afraid of having hypoglycaemia while you are alone?	4.4%	16.0%	16.7%	31.0%	31.9%
Do you fear not recognizing the symptoms of hypoglycaemia?	6.2%	12.7%	20.1%	27.8%	33.2%
Do you worry about losing consciousness due to hypoglycaemia?	5.8%	12.0%	15.2%	31.0%	35.9%
Are you afraid of falling asleep for fear of having hypoglycaemia at night?	11.8%	14.5%	16.3%	33.2%	24.1%
Are you afraid of having hypoglycaemia at work?	7.8%	14.2%	17.2%	34.7%	26.1%
Are you afraid of having hypoglycaemia outside of a health care setting?	8.9%	12.3%	16.5%	33.2%	29.0%
Are you afraid of not knowing what to do in the event of hypoglycaemia?	9.4%	14.2%	14.7%	33.9%	27.8%
Avoidance					
Do you avoid social situations (meetings, outings, etc.) due to fear of having a hypoglycaemic episode?	17.6%	18.0%	13.2%	29.2%	22.0%
Are you afraid of taking a trip/holiday for fear of experiencing hypoglycaemia?	15.6%	16.3%	16.3%	29.0%	22.7%
Do you stop doing things you used to do for fear of having a hypoglycaemic episode?	14.2%	15.2%	15.2%	32.8%	22.5%
Interference					
Do you have hypoglycaemia that makes you unable to work?	14.7%	15.8%	15.8%	32.5%	21.2%
Do you have hypoglycaemia that interferes with your family life?	13.4%	16.3%	16.0%	33.2%	21.1%
Do you have hypoglycaemia that makes you unable to drive or use machinery?	16.0%	13.8%	20.5%	31.8%	18.0%
Do you have hypoglycaemia that interferes with your social life?	14.7%	17.2%	15.2%	33.9%	18.9%
Do you have hypoglycaemia that interferes with your leisure activities?	12.5%	16.0%	15.2%	33.8%	22.5%

Predictors of poor mental wellbeing, problem areas in diabetes, and fear of hypoglycaemia

Binary logistic regression was used to identify predictors of poor mental wellbeing, problem areas in diabetes, and fear of hypoglycaemia. Three independent regression models were conducted to identify the predictors of the three outcome measures. 

Patients aged 31-35 years, those with a duration of diabetes (6-10 years), those who are on insulin therapy, and those who have reported hospital admission for hypoglycaemic episodes in the previous six months were more likely to have poor mental wellbeing status compared to others (p<0.05). Patients aged 31-35 years and 41-45 years, those with a duration of diabetes (6-10 years), and those who have reported hospital admission for hypoglycaemic episodes in the previous six months were more likely to have diabetes-related emotional distress compared to others (p<0.01). Patients aged 31-35 years, those with a duration of diabetes (6-10 years), and those who have reported hospital admission for hypoglycaemic episodes in the previous six months were more likely to have fear of hypoglycaemia compared to others (p<0.05). For further details, refer to Table 5.

**Table 5:** Predictors of poor mental wellbeing, problem areas in diabetes, and fear of hypoglycaemia. *p<0.05, **p<0.01, ***p<0.001, CI: confidence interval

Demographics	Odds ratio of having poor mental wellbeing status (95% CI)	Odds ratio of having diabetes-related emotional distress (95% CI)	Odds ratio of having fear of hypoglycaemia (95% CI)
Gender			
Male (Reference category)	1.00	1.00	1.00
Female	1.03 (0.73-1.46)	0.89 (0.63-1.25)	1.11 (0.72-1.71)
Age			
18-25 years (Reference category)	1.00	1.00	1.00
26-30 years	1.36 (0.86-2.13)	1.23 (0.79-1.90)	1.53 (0.85-2.76)
31-35 years	3.45 (1.95 – 6.13)***	2.00 (1.23-3.25)**	2.35 (1.18-4.67)*
36-40 years	1.13 (0.64 – 2.00)	1.48 (0.82-2.68)	1.44 (0.66-3.12)
41-45 years	0.95 (0.51-1.76)	2.05 (1.00-4.19)*	1.08 (0.49-2.36)
46-50 years	0.77 (0.40-1.48)	1.20 (0.60-2.40)	0.71 (0.33-1.53)
51 years and over	0.42 (0.27 – 0.65)***	0.30 (0.20-0.47)***	0.32 (0.20-0.52)***
Marital status			
Unmarried (Reference category)	1.00	1.00	1.00
Married	0.77 (0.53-1.11)	1.10 (0.77-1.57)	0.63 (0.39-1.02)
Educational level			
Not educated (Reference category)	1.00	1.00	1.00
Completed primary or lower	0.74 (0.35-1.59)	0.52 (0.25-1.10)	0.79 (0.31-1.97)
Completed secondary grade	0.82 (0.56-1.19)	0.82 (0.56-1.20)	0.83 (0.52-1.32)
College/university or above	1.06 (0.75-1.51)	1.27 (0.89-1.79)	1.16 (0.75-1.79)
Employment status			
Unemployed or retired (Reference category)	1.00	1.00	1.00
Employed	0.88 (0.62-1.25)	1.24 (0.88-1.76)	1.33 (0.86-2.07)
Student	0.77 (0.49-1.22)	0.96 (0.61-1.51)	1.74 (0.89-3.39)
Type of diabetes mellitus			
Type 1 diabetes mellitus (Reference category)	1.00	1.00	1.00
Type 2 diabetes mellitus	0.70 (0.47 – 1.04)	0.44 (0.29-0.67)***	0.60 (0.35-1.02)
Duration of the disease			
Less than one year (Reference category)	1.00	1.00	1.00
Between 1 and 5 years	0.89 (0.61-1.31)	0.86 (0.59-1.26)	1.41 (0.85-2.35)
Between 6 and 10 years	1.97 (1.28-3.03)**	2.42 (1.57-3.75)***	1.94 (1.11-3.38)*
Between 11 and 15 years	1.08 (0.66-1.75)	1.64 (0.98-2.74)	0.95 (0.53-1.71)
More than 15 years	0.67 (0.43-1.03)	0.59 (0.38-0.90)*	0.53 (0.32-0.87)*
Diabetes medication regimen			
Oral antidiabetic medications (Reference category)	1.00	1.00	1.00
Insulin	1.57 (1.04-2.36)*	1.36 (0.92-2.02)	1.22 (0.74-2.01)
Combination (Oral medications and insulin)	1.15 (0.74-1.78)	1.41 (0.90-2.20)	0.93 (0.55-1.58)
Combination (oral medications and other injectable antidiabetic agents)	1.20 (0.63-2.28)	1.47 (0.76-2.82)	2.53 (0.89-7.19)
Hospital admission for hypoglycaemic episodes in the previous 6-months			
No (Reference category)	1.00	1.00	1.00
Yes	4.26 (2.87-6.33)***	4.18 (2.84-6.13)***	5.22 (3.01-9.06)***
Did the patient experience a drop in the blood sugar level, but did not require him to go to the hospital and was able to deal with it alone or with the help of those close to him?			
No (Reference category)	1.00	1.00	1.00
Yes	1.31 (0.92-1.85)	1.18 (0.84-1.67)	0.86 (0.56-1.33)

## Discussion

In this study, the mental wellbeing and the quality of life of patients with diabetes and suffering from hypoglycaemia in Saudi Arabia were investigated. The majority of the study participants (85.1%) reported that they have experienced hypoglycaemic events. Around (45.9%) reported that they were admitted to the hospital for hypoglycaemic episodes in the previous six months, with around (54.9%) reported experiencing a drop in the blood sugar level but did not require them to go to the hospital. A previous study that investigated the prevalence of hypoglycaemia among patients with diabetes in Saudi Arabia reported that around 52% of the study participants reported that they had at least one hypoglycaemic attack [17]. This result is consistent with our results. However, it is important to mention that this study was conducted on patients during the month of Ramadan where Muslim individuals usually fast meaning that they are at higher risk of hypoglycaemia due to changes in their diet [17]. Another cross-sectional survey study that was also conducted in Saudi Arabia reported that the prevalence of diabetic coma among the studied diabetic patients was 57.5% and that the type of coma was mainly hypoglycaemia in 70.7% of the study participants [18]. These results are also similar to the results reported in our study.

In our study, around 69.7% of the study participants had a low score (below 13) on the WHO-5 scale indicating poor mental wellbeing status, possible depression, and reduced quality of life. Additionally, only a minority of study participants (2.6-4.5%) reported being free of any mental health problem. A previous study that included around 17,000 patients with areal-world data from Germany reported that 27.0% of the study sample were likely to have depression according to the WHO-5 scale [19]. Our study reported almost double of these results. However, it is important to highlight that depression is also a multifactorial disease and that several factors could initiate symptoms of depression [24]. The mental status of patients with diabetes is hugely affected by multiple factors including restriction of diet, lifestyle modifications, polypharmacy and anxiety and depression about the complications of the diseases as well [25-27].

Patients with diabetes usually suffer from mental and emotional issues which can affect the quality of life of these patients. These issues are likely to be due to disease and treatment-related complications including hypoglycaemia [28]. In our study, around 67.8% of the study participants reported that they have some possible diabetes-related emotional distress, with around 89.1% of the study participants reported being worried about the future and complications of diabetes. In addition, around 90.0% of the study sample had issues in coping with the complication of diabetes. These results were consistent with previous studies reporting that diabetes micro- and macrovascular complications have a negative impact on patients' quality of life [29,30]. Patients with diabetes are likely to have an increased risk of morbidity and mortality. Some of these complications may hugely affect the quality of life of patients with diabetes such as cardiac, neurological and nephrological complications [31,32].

Our study also showed that most of the study participants (83.4%) expressed fear of hypoglycaemia. Hypoglycaemic patients may experience fear and anxiety about future hypoglycaemic events, putting them in fear and distress. This was highlighted in a previous qualitative study that reported that hypoglycaemic concerns are significant in patients with diabetes [33]. In addition, a previous study that was conducted in Saudi Arabia reported an increased fear of hypoglycaemia among adolescents patients with diabetes [34], with similar other reviews reporting that fear of hypoglycaemia remains a major issue among patients with diabetes [35]. Due to the unpleasant aspects of hypoglycemia, diabetic patients become anxious and concerned about having recurrent and frequent hypoglycaemic events [23]. This might lead to changes in their treatment plan and considering less intensive antidiabetic therapy [4]. Additionally, diabetic patients at higher risk of developing hypoglycaemia might adopt over-compensatory behaviours in order to avoid the aversive symptoms of hypoglycaemic events, including treatment nonadherence (taking lower insulin dose) or skipping it [36].

Our results showed that patient's aged 31-35 and 41-45 years, with diabetes for 6-10 years, on insulin therapy, and hospitalized for hypoglycaemic episodes in the preceding six months were more likely to have poor mental wellbeing, diabetes-related emotional distress, and have fear of hypoglycaemia. These findings are consistent with previous research indicating that patients with more chronic diseases and more severe diseases are at an increased risk of developing poor mental health and quality of life [37]. Moreover, the study results corroborate prior research indicating that patients with younger age at diagnosis, longer duration of diabetes, and insulin therapy are more likely to develop poor mental health and quality of life [17,19].

Doctors and health care professionals must be aware of such results and more attention to hypoglycaemic events and complications must be made [38]. Public health awareness and preventions strategies must be provided for patients with diabetes mellitus.

This study has several strengths. First, it is the first study in Saudi Arabia that investigated the mental health status and the quality of life of patients with diabetes mellitus suffering from hypoglycaemia. Secondly, in this study, we used three validated scales to investigate the study's objective, which increased the reliability of our findings. This study, however, has some limitations. First, the study is a cross-sectional survey, which limits our ability to control for multiple factors. Second, our study sample was recruited through an online survey. However, our data collection occurred during the COVID-19 pandemic, during which the majority of Saudi citizens worked virtually and online. As a result, it is reasonable to assume that this will not affect the overall conclusion.

## Conclusions

Depressive symptoms and reduced quality of life are common among patients with diabetes mellitus suffering from hypoglycaemia in Saudi Arabia. Future studies to investigate the factors associated with these symptoms are needed.
